# Income level and chronic ambulatory care sensitive conditions in adults: a multicity population-based study in Italy

**DOI:** 10.1186/1471-2458-9-457

**Published:** 2009-12-11

**Authors:** Nera Agabiti, Monica Pirani, Patrizia Schifano, Giulia Cesaroni, Marina Davoli, Luigi Bisanti, Nicola Caranci, Giuseppe Costa, Francesco Forastiere, Chiara Marinacci, Antonio Russo, Teresa Spadea, Carlo A Perucci, Italian Study Group on Inequalities in Health Care

**Affiliations:** 1 Epidemiology Department, Local Health Authority RM/E, Rome, Italy; 2 Modena Cancer Registry, Modena, Italy; 3 Epidemiology Department, Local Health Authority; Milan, Italy; 4 Regional Agency for Health and Social Care - Emilia Romagna Region, Bologna, Italy; 5 Epidemiology Department, Piedmont Region, Turin, Italy; 6 Epidemiology Department, Local Health Authority, La Spezia, Italy

## Abstract

**Background:**

A relationship between quality of primary health care and preventable hospitalizations has been described in the US, especially among the elderly. In Europe, there has been a recent increase in the evaluation of Ambulatory Care Sensitive Conditions (ACSC) as an indicator of health care quality, but evidence is still limited. The aim of this study was to determine whether income level is associated with higher hospitalization rates for ACSC in adults in a country with universal health care coverage.

**Methods:**

From the hospital registries in four Italian cities (Turin, Milan, Bologna, Rome), we identified 9384 hospital admissions for six chronic conditions (diabetes, hypertension, congestive heart failure, angina pectoris, chronic obstructive pulmonary disease, and asthma) among 20-64 year-olds in 2000. Case definition was based on the ICD-9-CM coding algorithm suggested by the Agency for Health Research and Quality - *Prevention Quality Indicators*. An area-based (census block) income index was used for each individual. All hospitalization rates were directly standardised for gender and age using the Italian population. Poisson regression analysis was performed to assess the relationship between income level (quintiles) and hospitalization rates (RR, 95% CI) separately for the selected conditions controlling for age, gender and city of residence.

**Results:**

Overall, the ACSC age-standardized rate was 26.1 per 10.000 inhabitants. All conditions showed a statistically significant socioeconomic gradient, with low income people being more likely to be hospitalized than their well off counterparts. The association was particularly strong for chronic obstructive pulmonary disease (level V low income vs. level I high income RR = 4.23 95%CI 3.37-5.31) and for congestive heart failure (RR = 3.78, 95% CI = 3.09-4.62). With the exception of asthma, males were more vulnerable to ACSC hospitalizations than females. The risks were higher among 45-64 year olds than in younger people.

**Conclusions:**

The socioeconomic gradient in ACSC hospitalization rates confirms the gap in health status between social groups in our country. Insufficient or ineffective primary care is suggested as a plausible additional factor aggravating inequality. This finding highlights the need for improving outpatient care programmes to reduce the excess of unnecessary hospitalizations among poor people.

## Background

The quality of primary heath care (PHC) is a key element in effective and efficient health systems. Primary care includes multiple and diverse services that are difficult to measure and information is not adequate to construct standardized indicators of quality at that level. In recent years a method has been developed using secondary databases to determine how successfully PHC deals with health problems[1,2]. The hospitalization rate for Ambulatory Care Sensitive Conditions (ACSC) is one of these indicators, and represents the volume of hospital activity potentially preventable by timely and effective primary care. Several studies have documented a relationship between greater access to PHC and lower risk for preventable hospitalizations [3-5]. Angina, asthma, chronic obstructive pulmonary disease (COPD), hypertension, congestive heart failure (CHF) and diabetes account for most chronic ACSC hospitalizations.

Mechanisms of socioeconomic disparity in health encompass differential exposure to behavioural and environmental risk factors, however inhomogeneous access to primary health services may play a critical role [6-8]. Several studies from the US describe variability in ACSC hospitalizations. Racial and ethnic minorities are more likely than Caucasians to be hospitalized for ACSC, [9-11] and the rates of hospitalisation for ACSC are higher for people with lower levels of education or income,[7,12] residents in rural areas,[13,14] uninsured [11,15] and Medicare beneficiaries[11,14]. The validity of such an indicator has been tested in China,[16] New Zealand,[17] Australia,[18] Canada,[7], but in Europe it has been evaluated only in studies conducted in the Spanish Health System[3,8,19]. There is still a need for information from European countries with health care systems that differ from that in the US (health insurance and restricted public funding)[1].

In Italy there is a universal and comprehensive publicly-funded health care system; the responsibility is shared by the State and the 20 Regions, with large differences in health service organization and provision. The rationale behind this system is to offer free health care to all, without bias, regardless of financial or other personal resource. However there is growing evidence that socioeconomic disparities do alter access to effective-appropriate treatments across all major medical and surgical conditions: low socioeconomic individuals are vulnerable and tend to receive substandard care[20,21]. Limited access to primary prevention interventions or treatments (i.e. screening programs for cancer, dental examinations or vaccine immunizations in children) among disadvantage people has been recently highlighted by the latest National Health Interview Survey conducted by the National Center for Health Statistics[22]. Only two studies on ACSC have been conducted in our country - one from the city of Bologna based on population discharge abstract datasets and the other in an area of Southern Italy with a medical chart review methodology for about 500 hospitalized patients: they supported the usefulness of identifying avoidable hospitalizations to evaluate quality of health care in our country[23,24]. We conducted a multicity population-based study to determine whether income level is associated with hospitalization rates for ACSC in adults.

## Methods

### Study Population

Data of city residents in the year 2000 were extracted from the population register of each participating city: Rome (about 2.8 million inhabitants), Bologna (about 400,000 inhabitants), Turin (about 900,000 inhabitants) and Milan (about 1.4 million inhabitants).

### Hospital Admissions for ACSC

From the Hospital discharge data Information System (HIS) we selected all acute hospital discharges between the 1^st ^of January and the 31^st^of December 2000 for people aged 20-64 years who were diagnosed with any of the following six chronic conditions: *diabetes, hypertension (without procedures), congestive heart failure (CHF) (without procedures), angina pectoris (without procedures), chronic obstructive pulmonary disease (COPD), and asthma*. We excluded admissions to rehabilitation wards and admissions outside the region of residence. The conditions were identified from specific ICD-9-CM codes registered as the main diagnosis on the basis of the coding algorithm for Prevention Quality Indicators recently developed by the Agency for Health Care Research and Quality[25]. Following a validated ICD-9-CM coding algorithm we identified selected comorbidities including cardiac and circulatory disease, vascular disease (including cerebrovascular), hypertension, pulmonary disease, renal disease, liver disease, tumours in the hospitalized cohorts [26]. [see Additional file 1 part A].

### Indicator of Socioeconomic Status

As an indicator of individual socioeconomic status we used the 1998 city-specific index based on the median per capita income within each census block of residence. Details on the index have been reported elsewhere[20] Briefly, data relative to income earned in 1998 (Italian Tax Register) were linked to the Population Registers of the four cities to connect family information to each resident's income data and to calculate the net family income. We obtained the per capita income, weighted for the number of family members, and aggregated data at the census block level to calculate the median income. The median number of inhabitants per census block was 260. In order to obtain categorical values for the income indicator we calculated the quintiles of the income distribution by census block, for each city.

### Data analysis

The rate of hospitalization for the six ACSC was calculated by dividing the number of subjects with a hospital admission within a given income quintile by the corresponding population in the same socioeconomic group. All rates were directly standardised for gender and age (5-year age groups) to the Italian population and expressed as the number of hospitalizations per 10.000 inhabitants. Rate ratios were estimated with Poisson regression to assess the relationship between income level and hospitalization rates (RR, 95% CI). The patient characteristics considered in the Poisson analysis were age (20-44, 45-64), gender, and city of residence. They were introduced as potential confounders of the relationship under study. Quintiles of income were considered as a categorical variable (level I high income, level V low income), but p-values for linear trend were also calculated. Datasets were prepared using SAS 8.0, and all statistical analyses were performed using STATA software, version 8.2. All p-values reported are two-sided.

## Results

We analysed a total of 9.384 hospitalisations. The majority were men (61%) and resident in Rome (58%). Mean age was 54 years (SD 9.4). The lowest median family equivalent income was in Rome (12.204 Euro), the highest in Bologna (13.507 Euro). Overall, the age standardized ACSC rate was 26.1 per 10.000 inhabitants (Table 1): the lowest was observed in Turin (21.5), the highest in Rome (29.4).

**Table 1:** Descriptive characteristics and hospitalisation discharge rates of the Italian four cities - Adults 20-64 ages. Year 2000

			**Total hospitalisation rates**^†^		ACSC Hospitalisation rates†	
			
City of residence	Adult people 20-64 yrs of age (number)	Median family equivalent income (euro)	rate	95% CI	rate	95% CI
Turin	586.419	13.161	695,0	688.6-701.4	21,5	20.4-22.6
Rome	1.800.581	12.204	856,9	852.9-861.0	29,4	28.6-30.2
Milan	764.102	13.163	758,3	752.5-764.1	22,1	21.1-23.2
Bologna	240.025	13.507	825,4	814.6-836.2	27,3	25.3-29.3
						
Overall	3.391.127	12.947	804,4	801.6-807.3	26,1	25.6-26.6

^† ^Age and gender standardised rates per 10,000 inhabitants and 95% CI

Table 2 and Table 3 present the association between income level and ACSC hospitalization overall, and separately for the six conditions. In the whole study population, we found an inverse relationship between income level and hospitalisation rates; the lower the income, the higher the ACSC hospitalization rate (overall, level V low income vs. level I high income RR = 2.59, 95% CI: 2.35-2.85). There was a clear inverse gradient between income level and ACSC rates for all conditions. The strongest relative risk was found for COPD (level V vs. level I RR = 4.23, 95% CI: 3.37-5.31), the lowest for angina (level V vs. level I RR = 1.97, 95% CI: 1.70-2.30). Tests for linear trend were statistically significant overall and for each condition. The rates were higher among 45-64 years old aged individuals than in younger people (20-44 years of age). For all conditions except asthma, males were more likely to have ACSC hospitalization than females. Diabetes and CHF had similar rates in all cities studied. Compared to residents of Turin, residents of Rome showed higher rates for hypertension, angina, COPD and asthma, while residents of Milan higher rates for angina and those from Bologna for angina and COPD. In the overall population, comorbidity status slightly varied across income groups: no comorbidity 60% level I high income, 51% level V low income; one comorbidity 30% level I, 33% level V; two or more comorbidities 10% level I, 16% level V). [see Additional file 1 part B].

**Table 2:** Age standardised rates (per 10,000 inhabitants) and Rate Ratios (RR, 95% CI) for selected chronic conditions - Adults ages 20-64. Year 2000

	Diabetes				Hypertension (without procedures)				Congestive Heart Failure (without procedures)				Angina (without procedures)			
N			1.648				1.546				1.321				2.423	
rate (95%CI)			4,6	4.4-5.0			4,3	4.1-4.5			3,6	3.4-3.8			6,7	6.4-7.0

**Characteristics**																
	**%**	**rate**	**RR**	**95% CI**	**%**	**rate**	**RR**	**95% CI**	**%**	**rate**	**RR**	**95% CI**	**%**	**rate**	**RR**	**95% CI**

**Gender**																
Female	45,4	4,0	1,00		47,5	3,9	1,00		32,8	2,2	1,00		27,0	3,4	1,00	
Male	54,6	5,3	1,33	1.18-1.51	52,5	4,8	1,27	1.11-1.47	67,2	5,2	2,26	2.01-2.53	73,0	10,4	3,05	2.72-3.42
**Age group (years)**																
20-44	19,7	1,6	1,00		16,7	1,3	1,00		7,0	0,5	1,00		6,5	0,8	1,00	
45-64	80,3	9,1	5,75	5.00-6.61	83,3	8,8	7,42	6.32-8.72	93,0	8,3	19,36	15.7-23.9	93,5	15,4	21,21	17,88
**Income (quintiles)**																
I high	13,6	3,0	1,00		16,7	3,4	1,00		9,4	1,6	1,00		14,8	4,6	1,00	
II	14,2	3,2	1,08	0.87-1.34	17,0	3,5	1,00	0.80-1.26	16,4	2,9	1,80	1.44-2.24	19,6	6,3	1,33	1.13-1.57
III	18,1	4,2	1,46	1.19-1.80	19,4	4,2	1,19	0.95-1.48	18,9	3,4	2,18	1.76-2.71	19,9	6,7	1,43	1.21-1.68
IV	20,5	4,9	1,68	1.37-2.07	21,5	4,8	1,33	1.06-1.68	24,8	4,7	2,97	2.41-3.65	20,4	7,1	1,53	1.31-1.79
V low	33,6	8,4	2,77	2.29-3.36	25,4	5,9	1,64	1.31-2.04	30,4	6,0	3,78	3.09-4.62	25,3	9,3	1,97	1.70-2.30
*linear test for trend*				*< 0.001*								*< 0.001*				*< 0.001*
**Centre**																
Turin	16,2	4,3	1,00		8,7	2,1	1,00		18,6	3,8	1,00		14,7	5,6	1,00	
Rome	51,9	4,7	1,08	0.92-1.28	77,2	6,5	3,07	2.51-3.74	49,4	3,5	0,92	0.80-1.07	53,2	7,0	1,26	1.10-1.44
Milan	25,0	5,0	1,19	0.99-1.42	10,2	1,9	0,91	0.71-1.17	23,8	3,6	1,00	0.84-1.18	23,3	6,4	1,23	1.07-1.43
Bologna	6,9	4,4	1,04	0.82-1.32	3,9	2,3	1,09	0.80-1.50	8,2	4,1	1,10	0.88-1.38	8,8	8,0	1,47	1.23-1.76

**Table 3:** Age standardised rates (per 10,000 inhabitants) and Rate Ratios (RR, 95% CI) for selected chronic conditions - Adults ages 20-64. Year 2000

	COPD				Asthma				Overall			
N			1.764				682				9.384	
rate (95%CI)			4,9	4.6-5.1			2,0	1.8-2.1			26,1	25-6-26.6

**Characteristics**												
	**%**	**rate**	**RR**	**95% CI**	**%**	**rate**	**RR**	**95% CI**	**%**	**rate**	**RR**	**95% CI**

**Gender**												
Female	37,9	3,5	1,00		62,6	2,4	1,00		39,1	19,4	1,00	
Male	62,1	6,4	1,76	1.54-2.01	37,4	1,5	0,62	0.53-0.73	60,9	33,6	1,73	1.63-1.84
**Age group (years)**												
20-44	11,6	1,0	1,00		49,6	1,7	1,00		14,7	6,9	1,00	
45-64	88,4	10,6	10,96	9.24-13.0	50,4	2,4	1,42	1.22-1.65	85,3	54,6	8,42	7.86-9.03
**Income (quintiles)**												
I high	9,6	2,2	1,00		13,1	1,3	1,00		13,0	42,3	1,00	
II	14,2	3,3	1,40	1.09-1.80	15,8	1,5	1,22	0.92-1.62	16,5	28,5	1,26	1.14-1.40
III	19,9	4,8	1,97	1.53-2.53	20,5	2,1	1,62	1.25-2.12	19,4	25,3	1,54	1.40-1.71
IV	19,4	4,8	2,13	1.68-2.71	21,7	2,2	1,73	1.33-2.25	21,1	20,7	1,75	1.59-1.94
V low	36,9	9,7	4,23	3.37-5.31	28,9	3,0	2,37	1.84-3.04	29,9	15,9	2,59	2.35-2.85
*linear test for trend*				*< 0.001*				*< 0.001*				
**Centre**												
Turin	15,1	4,1	1,00		14,4	1,7	1,00		14,6	21,5	1,00	
Rome	58,4	5,6	1,38	1.15-1.66	56,7	2,1	1,29	1.03-1.61	57,6	29,4	1,38	1.27-1.49
Milan	16,6	3,4	0,90	0.73-1.10	21,9	1,9	1,17	0.90-1.51	20,1	22,1	1,09	0.99-1.18
Bologna	9,9	6,6	1,65	1.34-1.97	7,0	2,0	1,20	0.85-1.70	7,7	27,3	1,29	1.16-1.44

## Discussion

This study provides evidence of higher rates of hospitalization for ACSC for economically disadvantaged people in Italy, where barriers to health care are not expected to exist because of the universal health care system. The magnitude of the effect differs according to specific conditions, and was most relevant for COPD and CHF. Males seem more vulnerable than females to hospitalization for all conditions studied except asthma.

Hospitalization for ACSC has been extensively used as an indicator of the accessibility and effectiveness of PHC in the US, where governments use it to plan evidence-based intervention and control health care costs. On the contrary, this practice has not been widely adopted in Europe. Several studies support the validity of ACSC as an indicator of access barriers and a possible outcome measure for primary-care services, since many of these conditions can be avoided with timely and effective treatment and heath education provided by PHC[2,3,7,5,8]. Poor access to primary care is associated with higher ACSC in universal health insurance medical care systems, like in Australia[18]. In the US, rural residents tend to postpone access to needed care and their risk of hospitalization increase;[14] more primary care physicians or public ambulatory clinics have been associated with better access to care and lower ACSC hospitalization rates[4,18]. On the other hand, in Canada where no formal barrier to primary care exists, residents of the lowest income neighbourhoods had higher hospitalization rates for selected ACSC than those of their counterparts from the highest income areas controlling for the number of ambulatory visits [7].

The debate continues on the validity of ACSC hospitalizations as an indicator of PHC[1]. Actually, it is likely that there are a variety of interrelating factors associated with hospitalization for ACSC. They include socioeconomic and demographic characteristics of the population, ACSC prevalence, and availability of primary care or alternative health care services[3,19]. In a study exploring factors related to ACSC hospitalization, accessibility to hospital was the only variable associated with higher rates, while variability in admission rates for ACSC was not associated with primary care characteristics[19]. Potential biases in using admission rates as a measure of PHC performance have been highlighted: ACSC hospitalization rates mainly reflect disease prevalence, increased disease severity and multiple comorbidities and they do not strictly depend on the quality of primary care[7,27]. The weak socioeconomic gradient observed in the prevalence of chronic conditions among Italian 20-64 year-old people - as a result from the Italian National Health Interview Survey (e.g. prevalence of "at least one chronic disease" was 19% for well off and 25% for disadvantaged people in 40-45-year age group; the corresponding figures in 65-69-year age group were 42% and 63% [22] - and the relative small differential in comorbidity status in our study suggest further mechanisms, other than different health status, involved in the observed social gap of ACSC admission rates. Moreover, a good and equitable PHC should reduce rather than increase income inequality in health, and we should observe lower ACSC hospitalization rates among poor people. Other studies support the hypothesis that attributing ACSC hospitalization rates exclusively to PHC services is not appropriate: they may depend, at least in part, on hospital admission policies and on the availability of hospital services in the geographical area of residence[4,6,8]. In this respect, we analysed the city of residence as a proxy of availability of primary care physicians and other health care professionals in the area, including inpatient and outpatient capacity, and confirmed previous findings on geographical variability in ACSC admissions rates[19].

Given the complex underlined mechanisms of the relationship under study, our analysis tried to take into account at least the main determinants of disease prevalence and hospitalization admissions i.e. gender and age): both have been found to influence quality of care in different settings as well[6,19,28]. The higher rates found in our study in older than in younger adults could be explained in part by the higher occurrence or severity of ACSC conditions in older adults confirming previous findings[6,19]. Similarly, the higher rates among males - consistent with other studies [8,9,12,17] - could reflect higher prevalence or severity of ACSC conditions. On the other hand, the higher rate of asthma hospitalizations among females was an unexpected result because asthma is more prevalent among males. In this case, the validity of the ACSC indicator as a measure of PHC performance is clear; in fact, our results confirm suggestions from previous studies that women receive worse asthma care than men[13,29].

The selection of diagnoses to be considered as ACSC is one of the most important aspects of the methodology. There have been various diagnostic codes for hospital discharges - of both acute and chronic conditions - proposed and validated for use as indicators of primary care intervention quality, starting with those introduced in the US in 1993 by the Institute of Medicine, and followed by Billings and other authors[30,31]. Caminal and colleagues provided a specific list of ACSC conditions to be used to analyse responsibility by PHC care and specialist care both in Spain and in other European contexts[3]. They suggested that the choice of ACSC should be country -specific, due to the wide variety of health systems in operation.

In our study, we examined the six most common chronic conditions included in almost all the studies about ACSC. We evaluated the ICD-9-codes adopted in the literature based on their internal validity and used the more recent *AHRQ - Guide to Prevention Quality Indicator *coding definition because it was the best choice to address the specific effectiveness of PHC in our country[25]. For these diagnostic codes, evidence exists that specific PHC interventions reduce hospitalizations rates[3,8]. The use of 5 digits allowed us to increase specificity and reduce the influence of hospital admission policies on ACSC rates. For example, among diabetic patients we identified only codes for acute or chronic complications requiring hospitalizations, assuming that they should be limited by effective monitoring and pharmaceutical treatment. Similarly, early diagnosis and appropriate treatment and follow-up - which are all within the domain of primary care - influence the volume of hospitalizations for re-exacerbations of heart failure. Lastly, in asthma and COPD patients, early detection and monitoring of acute episodes together with appropriate follow up should successfully reduce the need for hospital care.

A number of studies have demonstrated variability in health care access and quality of primary care across social groups, but their main focus was on racial/ethnic differences. In the US, black, Medicaid and uninsured people were more likely to experience emergency department visits for five common chronic conditions as an indicator of reduced access to primary care[32]. Among African Americans and Hispanics, the risk for preventable hospitalizations - such as for asthma, diabetes, hypertension - was higher than among whites[10,14,33]. Similar patterns of association were observed in other studies of minorities with regards to asthma, diabetes and other conditions in the US[16,29,34]. Racial disparities in quality of Medicaid Managed Care measured by specific indicators of appropriate primary care in eligible patients were present, though diminishing, in the US between 1997 and 2003[11]. As regards socioeconomic status, Billings et al in New York City in 1988 suggested that lack of timely and effective outpatient care may lead to higher hospitalization rates in low-income areas[30]. Low income and education were strong predictors of higher ACSC rates for five chronic conditions in adults living in urban areas in California[31]. In Canada, living in a low-socioeconomic neighbourhood was a marker of higher vulnerability to hospitalization or emergency department visits for diabetes complications that should be prevented by quality care in the ambulatory setting, especially for adults[12]. Similarly, a census-based small area socioeconomic deprivation index was associated with higher rates of potentially avoidable hospitalizations in New Zealand[17]. Non-whites, low-income, rural residents and people without a primary care physician had significantly more ACSC hospitalizations than their counterparts in South Carolina[13]. In a National Hospital Discharge Survey in the US in 1990, African-Americans and residents of middle and low income areas had higher rates of potentially avoidable hospitalizations[9]. It is notable that differences were not significant among over 65 year-old people. Living in lower-income area in Texas was associated with higher rates of preventable hospitalization[35] while in the Canadian universal health care setting, the poor had reasonable access to ambulatory care for ACSC[7].

In the present study we used an aggregate indicator of income that has already been applied in different settings[20]. In studies on ACSC characterizing socioeconomic status, using mean neighbourhood income generally seems appropriate,[9,13,29] even though attributing an aggregated indicator to an individual can underestimate the true association[36]. Our area-based income index refers to the material well-being and standards of living of all family members; it includes economic resources provided by work, pension, real estate and investments. However, income level might not reflect other conditions relevant for health or the propensity to seek health care, such as level of education or occupation[37]. Other studies have shown that the association between income and health persists after adjusting for individual variables,[38] and the predictive power of economic poverty indicators has been shown to be comparable to that of composite deprivation indices[39]. We consider our income indicator a good proxy for the complex construct of socioeconomic position as we found a correlation coefficient higher than 0.7 between the income index and more composite socioeconomic position indicators available both for Rome and Turin[40]. One last point is the possible misclassification of income level. Our indicator is based on reported income tax from 1998 and our tax system tends to register many reports of low incomes, because tax evasion is relatively common among the rich in Italy[41]. Our low-income level could have included rich people also: this misclassification of exposure could have led to an underestimate of the effect.

The population-based design and the choice of specific chronic ACSC based on a validated algorithm are the main strengths of this study. We excluded old people because relative disparities in health tend to be weaker in old age and in order to limit the potential confounding effect of disease prevalence and comorbidity status. Some limitations include the inability to take into account the different distribution of disease prevalence and severity - including the presence of comorbidities - across income-level groups, which may contribute to higher hospitalization rates[3,18,27]. Finally, access to PHC may involve a variety of factors including time costs to obtain appointments or spent in waiting rooms, travel time or distance to the nearest hospital, other transportation barriers, and the availability of evening-night or weekend appointments, availability of primary care physicians and other health care professionals in the area. Although unlikely, our study does not allow distinguishing specific mechanisms potentially involved in social disparities in PHC, particularly regarding its availability, accessibility, or appropriateness.

## Conclusions

This study highlights that disadvantaged people experience more need of hospital care care. Barriers to PHC are a plausible cause for the observed inequality but further research is required to verify this hypothesis and examine the complexity of economic, structural, and cultural factors that affect access to care.

## Competing interests

The authors declare that they have no competing interests.

## Authors' contributions

NA participated in the design of this study, in the planning of the analysis and interpretation of the results, and drafted the manuscript; MP participated in the design, performed the statistical analysis and helped to interpret the results and draft the manuscript; CAP, FF, PS, NC, CM, TS participated in the design and coordination of this study and helped to draft the manuscript; GC, MD, GC, LB, AR participated in the design and helped to interpret the results. All authors read and approved the final manuscript. The Italian Study Group on Inequalities in Health Care participated in the initial phase of the whole program at national level and in the data collection.

## Pre-publication history

The pre-publication history for this paper can be accessed here:

http://www.biomedcentral.com/1471-2458/9/457/prepub

## Supplementary Material

Additional file 1 **ICD-9-CM codes and comorbidity status by income level**. Details on ICD-9-CM codes used for cohort selection and information on comorbidity status by income level are presented. Click here for file
