# Supplementary material for: Income level and chronic ambulatory care sensitive conditions in adults: a multicity population-based study in Italy
Source: BMC Public Health. 2009 Dec 11;9:457. doi: 10.1186/1471-2458-9-457 (PMC2804615; doi:10.1186/1471-2458-9-457)
Supplement: Additional file 1 — ICD-9-CM codes and comorbidity status by income level. Details on ICD-9-CM codes used for cohort selection and information on comorbidity status by income level are presented. [file 1471-2458-9-457-S1.DOC]

**Additional file 1**

**ICD-9-CM codes and comorbidity status by income level**

**PART A - Selection criteria for ACSC hospitalizations**

**ICD-9-CM coding algorithm**

Diabetes: main diagnosis 25002, 25003, 25010, 25011, 25012, 25013, 25020, 25021, 25022, 25023, 25030, 25031, 25032, 25033, 25040, 25041, 25042, 25043, 25050, 25051, 25052, 25053, 25060, 25061, 25062, 25063, 25070, 25071, 25072, 25073, 25080, 25081, 25082, 25083, 25090, 25091, 25092, 25093, 2510

Hypertension: main diagnosis 4010, 4019, 40200, 40210, 40290, 40300, 40310, 40390, 40400, 40410, 40490

*Exclusion criteria*: procedure code any position 3500, 3501, 3502, 3503, 3504, 3510, 3511, 3512, 3513, 3514, 3520, 3521, 3522, 3523, 3524, 3525, 3526, 3527, 3528, 3525, 3526, 3531, 3532, 3533, 3534, 3535, 3539, 3541, 3542, 3550, 3551, 3552,3553, 3554, 3560, 3561, 3562, 3563, 3570, 3571, 3572, 3573, 3581, 3582, 3583, 3584, 3591, 3592, 3593, 3594, 3595, 3596, 3598, 3599, 3601, 3602, 3603, 3604, 3605, 3606, 3607, 3609, 3610, 3611, 3612, 3613, 3614,3615, 3616, 3617, 3619, 362, 363, 3631, 3632, 3639, 3691, 3699, 370, 3710, 3711, 3712, 3731, 3732, 3733, 3734, 3735, 374, 375, 3751, 3752, 3753, 3754, 376, 3761, 3762, 3770, 3771, 3772, 3773, 3774, 3775, 3776, 3777, 3778, 3779, 3780, 3781, 3782, 3783, 3785, 3786, 3787, 3789, 3794, 3795, 3796, 3797, 3798

Congestive Heart Failure: main diagnosis 40201, 40211, 40291, 40401, 40403, 40411, 40413, 40491, 40493, 4280, 4281, 4289

*Exclusion criteria*: procedure code any position 3500, 3501, 3502, 3503, 3504, 3510, 3511, 3512, 3513, 3514, 3520, 3521, 3522, 3523, 3524, 3525, 3526, 3527, 3528, 3525, 3526, 3531, 3532, 3533, 3534, 3535, 3539, 3541, 3542, 3550, 3551, 3552,3553, 3554, 3560, 3561, 3562, 3563, 3570, 3571, 3572, 3573, 3581, 3582, 3583, 3584, 3591, 3592, 3593, 3594, 3595, 3596, 3598, 3599, 3601, 3602, 3603, 3604, 3605, 3606, 3607, 3609, 3610, 3611, 3612, 3613, 3614,3615, 3616, 3617, 3619, 362, 363, 3631, 3632, 3639, 3691, 3699, 370, 3710, 3711, 3712, 3731, 3732, 3733, 3734, 3735, 374, 375, 3751, 3752, 3753, 3754, 376, 3761, 3762, 3770, 3771, 3772, 3773, 3774, 3775, 3776, 3777, 3778, 3779, 3780, 3781, 3782, 3783, 3785, 3786, 3787, 3789, 3794, 3795, 3796, 3797, 3798

Angina: main diagnosis 4111, 41181, 41189, 4130, 4131, 4139

*Exclusion criteria*: procedure code any position 3500, 3501, 3502, 3503, 3504, 3510, 3511, 3512, 3513, 3514, 3520, 3521, 3522, 3523, 3524, 3525, 3526, 3527, 3528, 3525, 3526, 3531, 3532, 3533, 3534, 3535, 3539, 3541, 3542, 3550, 3551, 3552,3553, 3554, 3560, 3561, 3562, 3563, 3570, 3571, 3572, 3573, 3581, 3582, 3583, 3584, 3591, 3592, 3593, 3594, 3595, 3596, 3598, 3599, 3601, 3602, 3603, 3604, 3605, 3606, 3607, 3609, 3610, 3611, 3612, 3613, 3614,3615, 3616, 3617, 3619, 362, 363, 3631, 3632, 3639, 3691, 3699, 370, 3710, 3711, 3712, 3731, 3732, 3733, 3734, 3735, 374, 375, 3751, 3752, 3753, 3754, 376, 3761, 3762, 3770, 3771, 3772, 3773, 3774, 3775, 3776, 3777, 3778, 3779, 3780, 3781, 3782, 3783, 3785, 3786, 3787, 3789, 3794, 3795, 3796, 3797, 3798

Chronic Obstructive Pulmonary Disease: main diagnosis 490, 4910, 4911, 49120, 49121, 4918, 4919, 4920, 4928, 494, 496; code 4660 main diagnosis associated with codes 491, 492, 494, 496 secondary diagnoses

Asthma: main diagnosis 49300, 49301, 49310, 49311, 49320, 49321, 49390, 4939

**PART B - TABLES ON COMORBIDITY STATUS BY INCOME LEVEL**
